# Deterministic secure quantum communication using a single *d*-level system

**DOI:** 10.1038/srep44934

**Published:** 2017-03-22

**Authors:** Dong Jiang, Yuanyuan Chen, Xuemei Gu, Ling Xie, Lijun Chen

**Affiliations:** 1 State Key Laboratory for Novel Software Technology, Nanjing University, Nanjing, 210046, P.R.China

## Abstract

Deterministic secure quantum communication (DSQC) can transmit secret messages between two parties without first generating a shared secret key. Compared with quantum key distribution (QKD), DSQC avoids the waste of qubits arising from basis reconciliation and thus reaches higher efficiency. In this paper, based on data block transmission and order rearrangement technologies, we propose a DSQC protocol. It utilizes a set of single *d*-level systems as message carriers, which are used to directly encode the secret message in one communication process. Theoretical analysis shows that these employed technologies guarantee the security, and the use of a higher dimensional quantum system makes our protocol achieve higher security and efficiency. Since only quantum memory is required for implementation, our protocol is feasible with current technologies. Furthermore, Trojan horse attack (THA) is taken into account in our protocol. We give a THA model and show that THA significantly increases the multi-photon rate and can thus be detected.

Quantum key distribution (QKD), invented by Bennett and Brassard^1^, is a technique for sharing secret keys between two distant legitimate users, Alice and Bob, such that the key is perfectly secret from any eavesdropper, Eve. Due to the nature of unconditional security, QKD has attracted intensive study, and many advanced works have been proposed over recent years^2^. The basis reconciliation step, however, wastes many photons, and QKD is thus plagued by low efficiency. For instance, since half of the photons are discarded in the basis reconciliation step, the theoretical efficiency of the BB84 protocol^1^ is only 50%, and the efficiency will be further reduced in the subsequent steps, such as parameter estimation, error correction, etc. To solve this problem, two branches of quantum communication, i.e., quantum secure direct communication (QSDC) and deterministic secure quantum communication (DSQC), were proposed in 2000^3^ and 2002^4^, respectively. In contrast to QKD, QSDC and DSQC can transmit a secret message between two legitimate parties without first generating a shared secret key^5^. The difference between QSDC and DSQC is that DSQC requires the legitimate parties to exchange at least one bit classical information to decode the secret message encoded in each photon, whereas QSDC does not^6^. Since these types of protocols significantly increase the efficiency of quantum communication, QSDC and DSQC have attracted intensive study, and many advancements have been achieved on both the theoretical and experimental sides.

In 2000, Long and Liu proposed the first QSDC protocol^3^. This work also, for the first time, noted that the transmission of quantum data should be performed in blocks (block transmission technique), which not only provided a clear way to design new QSDC protocols but also laid the foundation for the security of QSDC protocols. Following the above design pattern, Deng *et al*. put forward a QSDC protocol with Einstein-Podolsky-Rosen (EPR) pairs transmitted in blocks^7^. Deng *et al*. also proposed a single photon sequence-based QSDC protocol^8^. These protocols are feasible with current technologies, and they have already been demonstrated in experiments^9^,^10^. Refs 11 and 12 gave the first high-dimension superdense coding-based and Greenberger-Horne-Zeilinger (GHZ)-based QSDC protocols, respectively. In 2002, Beige *et al*. proposed the first DSQC protocol based on single photon transmission^4^. Li *et al*. put forward two DSQC protocols, one based on pure entangled states and the other on *d*-dimensional single photon states^13^. Zhu *et al*. gave a DSQC protocol based on a secret transmitting order of EPR pairs^14^. Wang *et al*. modified Zhu’s protocol by replacing an EPR pair with a single photon and proposed a DSQC protocol based on a secret transmitting order of single photons^15^. Lee *et al*. gave a GHZ-based DSQC protocol^16^, in which users can identify each other by checking the correlation of GHZ states. More recently, Liu *et al*. gave a DSQC protocol based on high-dimensional entanglement swapping^17^. Chang *et al*. proposed a DSQC protocol based on a three-particle W state and a quantum one-time pad^18^. Yuan *et al*. gave a novel scheme for DSQC using a three-qubit GHZ state^19^. Li *et al*. proposed a DSQC and authentication protocol based on an extended GHZ-W state and a quantum one-time pad^20^.

Clearly, existing QSDC and DSQC protocols fall into two categories: those based on entanglement and those based on single photon. Entanglement is one of the most interesting features in quantum mechanics, which plays a significant role in quantum communication. Therefore, intense theoretical efforts have been devoted to the generation of entangled states, and many remarkable achievements have been made experimentally. For example, Sackett *et al*. and Osnaghi *et al*. demonstrated entanglements in an ion trap^21^ and cavity QED^22^, respectively. Vaziri *et al*. demonstrated entanglement of qutrits utilizing the orbital angular momentum of photons^23^. Recently, refs 24 and 25 realized very high-dimensional entanglements. The developments in generating entangled states technologies laid the foundation for the implementation of entanglement-based QSDC and DSQC protocols, and many entanglement-based protocols have been demonstrated in experiments in recent years^9^. However, compared with single photon-based protocols, entanglement-based protocols have great disadvantages in scalability and feasibility. Therefore, single photon-based QSDC and DSQC have attracted more attention, and many schemes have recently been proposed.

In this paper, we propose a new DSQC protocol, which follows the design pattern proposed in ref. 3, using a photon sequence as the message carrier and transmitting quantum data in blocks. The order rearrangement technique proposed in ref. 26 and mutually unbiased bases (MUBs) are employed to ensure security and increase the efficiency. In our protocol, Alice randomly prepares a set of qudits and sends them to Bob (forward transmission), who uses some of the received photons to assess the security of the quantum channel. If the security is confirmed, Bob encodes his checking message and secret message on the remaining photons, disturbs the initial order of these photons, and sends them back to Alice (backward transmission). After Alice receives these photons, Bob publishes the position and order of the photons carrying the checking message. Alice measures these photons with the same basis when she prepares them to evaluate the security of the quantum channel. If the quantum channel is secure, Bob publishes the order of the remaining photons. By measuring these photons with the same basis as when she prepared them, Alice directly obtains Bob’s secret message. The security of the quantum channel is carefully checked in both forward and backward transmission. Any eavesdropping attack will affect the error rate and thus be detected. This guarantees the security of our protocol. The use of a higher dimensional quantum system makes our protocol achieve higher security and efficiency compared with existing single photon-based DSQC protocols. Additionally, Trojan-horse attack (THA) was taken into account in our protocol. We give a THA model and show that THA will significantly increase the multi-photon rate, leading to detection.

## Results

### Mutually unbiased bases

According to refs 27, 28, two orthogonal bases *B* and *B*′ of a *d*-dimensional Hilbert space are mutually unbiased if for 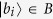 and 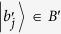 holds 
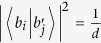
 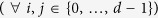. MUBs means a set of bases such that each two distinct bases are mutually unbiased. One can find *d* + 1 MUBs in *d* dimensions only if *d* is an odd prime. Besides the computational basis 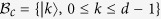, the explicit forms of the remaining *d* sets of MUBs are:


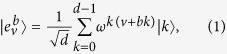


where 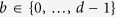 labels the basis, 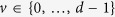 enumerates the vectors of the given basis, and 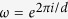 is the *d*^*th*^ root of unity. 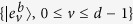 is a basis since for 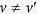



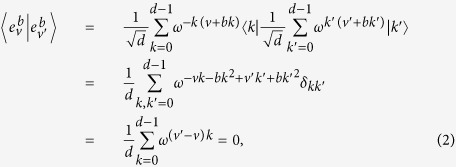


where 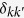 is the Kronecker’s delta. Equation (2) follows from 
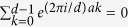
 for *a* ≠ 0. They are mutually unbiased since


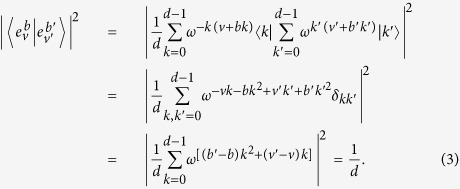


Equation(3) follows from the fact in number theory that 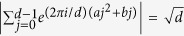 (*a* ≠ 0, *d* an odd prime).

### Protocol description

Since the complete sets of mutually unbiased bases are only known for dimensions which are powers of prime numbers, in our protocol, *d* is restricted to odd primes, and the following unitary operator is employed to manipulate photons


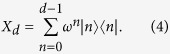


By applying *X*_*d*_, any vector 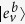 can be transformed into 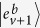. This is because that


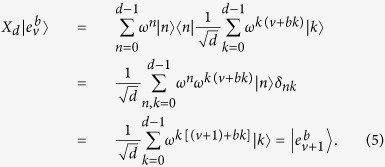


**Figure 1:**
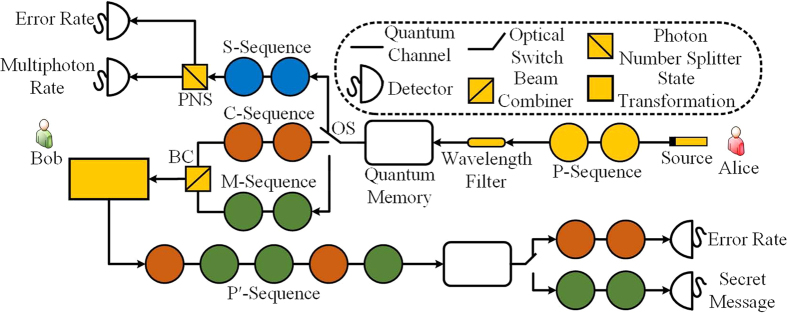
Workflow diagram of the presented QSDC protocol.

Now, let us describe our DSQC protocol in detail. If Bob wants to transmit a secret message to Alice, as shown in Fig. 1, our protocol runs as follows:

1. Alice prepares *n* single photons 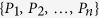 (*p*-sequence), each of which is prepared as follows: Alice randomly selects two numbers 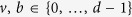 and prepares the state 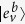. She then sends these photons to Bob.
2. Bob inserts a wavelength filter in front of his optical devices to filter out the photon signal with an illegitimate wavelength. Then he randomly selects a sufficiently large subset of photons (S-sequence), splits each sampling signal with a photon number splitter (PNS), and measures the two signals with two randomly selected bases. If the multi-photon rate or the error rate is unreasonably high, Alice and Bob abort the transmission. Otherwise, they continue to the next step.
3. Bob randomly chooses a sufficiently large subset of photons as a checking sequence (*C*-sequence). The remaining photons in the *P*-sequence form a message sequence (*M*-sequence). For each photon in the *C*-sequence, Bob randomly selects a number 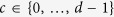 as the checking message, and encodes *c* on the photon by performing 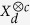. For each photon in the *M*-sequence, Bob encodes his secret message 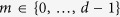 on the photon by performing 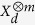.
4. By combining the *C*-sequence and the *M*-sequence, and disturbing the initial order of these photons, Bob constructs a rearranged photon sequence 
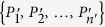
 (*P*′-sequence). He sends the *P*′-sequence to Alice. The rearranged order of the *P*′-sequence is completely secret to everyone but Bob.
5. After verifying that Alice has received the *P*′-sequence, Bob announces the position and the order of the *C*-sequence. For each photon in the *C*-sequence, Alice uses the same measuring basis as when she prepared the photon to measure it and reads out the checking message. Then, she publishes her results, and Bob can evaluate the error rate of the transmission. If the error rate exceeds the threshold, they abort the transmission. Otherwise, they continue to the next step.
6. Bob announces the rearranged order of the *M*-sequence. By performing the corresponding measurement on these photons according to their initial states, Alice obtains Bob’s secret message. For instance, if the initial state of the encoding photon is 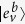, the result of the measurement is 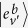, and Alice knows that Bob’s secret message is *m* = (*v*′ − *v* + *d*) mod *d*.

### Security and efficiency analysis

In this section, the security and efficiency of our protocol are analyzed. Our protocol is a two-way protocol that contains two transmissions of the quantum signal, i.e., from Alice to Bob (forward transmission) and from Bob to Alice (backward transmission). For some existing two-way protocols, such as the protocol proposed in ref. 29, because Alice sends one photon in one transmission, this protocol checks the security of the quantum channel while transmitting a secret message. This is unacceptable in practice since, when the error rate exceeds the threshold, part of the secret message may already be leaked. To solve this problem, the block transmission technique proposed in ref. 3 is employed in our protocol. By sending a block of photons in one transmission, the legitimate users can assess the security of the forward transmission before Bob encodes the checking message and the secret message. Similarly, in the protocol proposed in ref. 29, only the security of the forward transmission is checked. However, in a noisy channel, Eve can hide her eavesdropping in the noise. If Alice and Bob could not detect the eavesdropper in the forward transmission, the secret message would be partially or completely leaked. To solve this problem, the order rearrangement technique proposed in ref. 26 is employed in our protocol. By using the order rearrangement technique, the legitimate users can check the security of the backward transmission before Bob publishes the rearranged order of the *M*-sequence. Clearly, in our protocol the security of the forward and backward transmissions is carefully checked. Eve has to evade the security check conducted in both forward and backward transmissions to obtain the secret message. Otherwise, she can only obtain a set of meaningless data. Any eavesdropping attack will affect the error rate or the multi-photon rate and therefore be detected. Thus, the block transmission and order rearrangement techniques guarantee the security of our protocol. Next, we take two attack strategies as examples to provide a detailed analysis.

First, Eve may employ the intercept-resend attack in the hope that she can obtain some information about the secret message without being detected. The intercept-resend attack is one of the most practical attack strategies in quantum communications. In this attack, Eve intercepts the photon traveling from Alice to Bob, measures it with a randomly selected basis, and sends another photon prepared by herself to Bob according to the result of the measurement. After Bob performs his operation on this photon and sends it back, Eve intercepts the photon, uses the same measuring basis as when she prepared the photon to measure it, and reads out Bob’s operation. Since such an attack inevitably affects the error rate, it can be easily detected by measuring part of the photons to check the security of the quantum channel. For existing single photon-based DSQC schemes, since the photon is randomly prepared in one of two different bases, Eve can guess the correct basis with 50% probability. In this case, she cannot be detected at all. Otherwise, if Eve chooses the wrong basis, she still has a 50% probability to evade detection. Therefore, the probability of detecting Eve’s attack is 25%. This detection rate is quite low if the secret message is for some fatal event, such as the release of warheads^30^. Similarly, our protocol can detect such an attack in steps 2 and 5. However, in our protocol, since each photon is randomly prepared in one of *d* different MUBs, the detection rate *r*_*d*_ of our protocol is


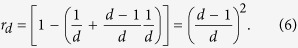


**Figure 2:**
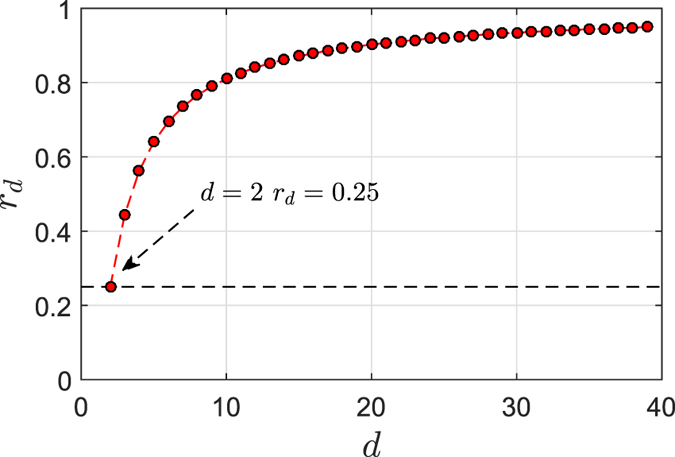
Detection rate *r*_*d*_ versus the dimensions *d*. The black dashed line indicates the detection rate of existing two-level quantum system-based schemes.

We plot the detection rate with respect to the dimensions *d* in Fig. 2. Clearly, the detection rate increases with the dimensions, and it would converge to 100% when *d* is large enough. This detection rate is far better than the detection rate of existing single photon based QSDC schemes, and clearly enables resistance to the intercept-resend attack.

**Figure 3:**
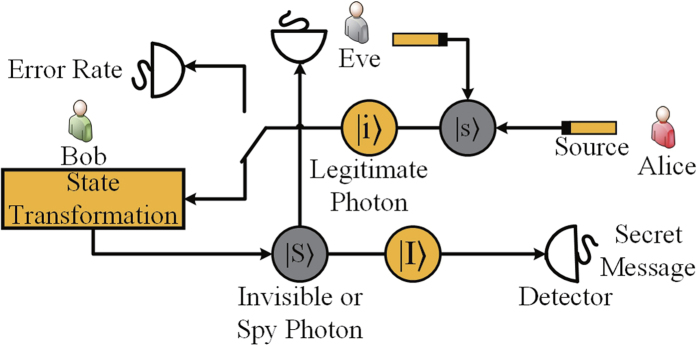
Workflow diagram of THA.

Another strategy that Eve can utilize to eavesdrop on Bob’s secret message is THA. In quantum cryptography, THA is a kind of attack strategy in which Eve can obtain some secret messages by sending signals that enter legitimate users’ devices through the quantum channel. The attack and defense strategies for QSDC and DSQC protocols were first discussed in ref. 5. This work gave two attack strategies, namely, the delay-photon THA and invisible photon THA. In the former strategy, as shown in Fig. 3, Eve can insert a spy photon in each signal prepared by Alice with a delay time shorter than the time windows of the detector. Then, she intercepts the signal traveling from Bob to Alice and separates the spy photon from the signal. She can obtain Bob’s operation by measuring the spy photon with the same basis as when she prepared the photon. The latter strategy uses the fact that the single photon detector is only sensitive to the photons with a special wavelength^5^. If the wavelength of a photon is far away from that used by Alice and Bob, this photon is invisible to the detector. Therefore, Eve can insert an invisible photon in each signal prepared by Alice and send it to Bob. When the signal is sent back from Bob, Eve intercepts the signal and separates the invisible photon from the signal. By measuring the invisible photon, Eve can read out Bob’s operation. Ref. 5 also describes two defense strategies. To resist the delay-photon THA, Bob adds a checking device that consists of a 50/50 photon beam splitter (PBS) and two single photon detectors. He can measure part of the received signals with this checking device. An unreasonably high multi-photon rate can indicate Eve’s attack. The invisible photon THA can be easily defeated by adding a wavelength filter before Bob’s optical devices. Existing DSQC schemes, such as those proposed in refs 14,15, cannot detect the THA. In these schemes, Alice and Bob check the security of the quantum channel by measuring part of the photons and calculating the error rate. However, since both the invisible photon and the spy photon do not click the detector, Eve’s attack will not affect the error rate. Eve, therefore, can break these schemes and obtain Bob’s secret message without being detected^5^.

In our protocol, by using the wavelength filter, Bob filters out the illegal photons before he handles the received signal. This guarantees resistance to the invisible photon THA. By using the checking device proposed in ref. 5, Bob can measure part of the legitimate signals and calculate the multi-photon rate which can be utilized to determine whether Eve exists. This defense strategy has been adopted by many quantum communication schemes to resist THA. However, to the best of our knowledge, none of them has discussed the relationship between the multi-photon rate and Eve’s attack. To fill this gap, we focus on modeling THA in this part. Since invisible photon THA can be easily resisted by adding a wave length filter, only delay photon THA is discussed. Generally, Eve has two attack strategies. First, to obtain as much of the secret message as possible, Eve inserts a spy photon in each legitimate signal. Obviously, if Alice has a perfect laser source, i.e., each legitimate signal contains only one photon, Bob can declare the presence of Eve once he detects two photon in one signal. Due to experimental imperfections, however, a legitimate signal may contain more than one photon. Suppose that the original multi-photon rate is 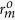, that is, 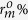 signals in the *P*-sequence contains multiple photons (for convenience of discussion, we assume that these signals can always be detected simultaneously by Bob’s two detectors). Also suppose that the *S*-sequence contains *n* signals. In these signals, there are 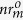 signals that can be detected simultaneously by Bob’s two detectors, regardless of the presence of Eve. Additionally, since Eve inserts a spy photon in each legitimate signal, there are 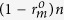 signals consisting of two photons. With 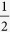 probability, these signals can be simultaneously detected by Bob’s two detectors. In total, Bob can detect 
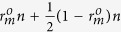
 signals that contain more than one photon. Therefore, the new multi-photon rate 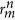 is


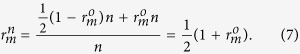


**Figure 4:**
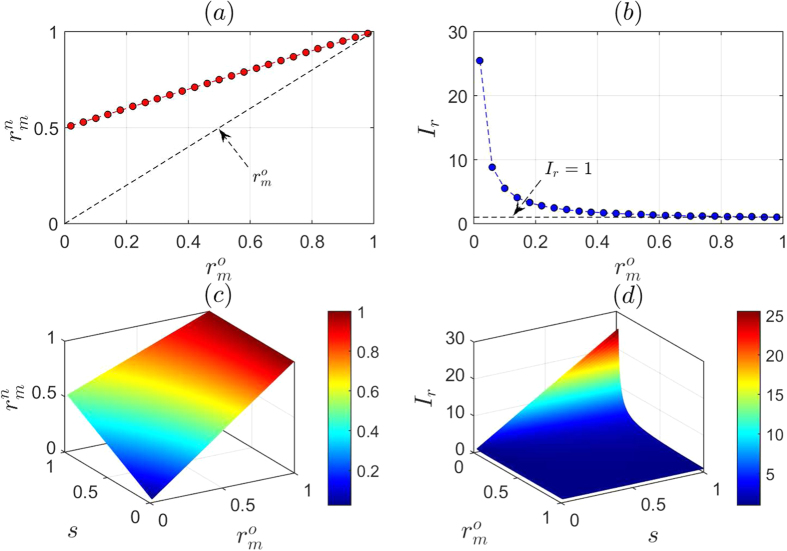
Evaluation of the multi-photon rate. (**a**) In the case that Eve inserts a spy photon in each signal, the multi-photon rate 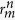 versus the original multi-photon rate 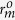. (**b**) Increase rate *I*_*r*_ of multi-photon rate versus 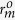. (**c**) In the case that Eve inserts a spy photon in a signal with probability *s*, 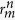 versus 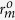 and *s*. (**d**) Increase rate *I*_*r*_ of multi-photon rate versus 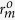 and *s*.

We plot the new multi-photon rate 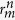 as a function of the original multi-photon rate 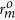 in Fig. 4(a). Clearly, 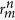 is significantly increased, particularly when the original multi-photon is small enough. As 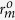 increases, however, the increase rate *I*_*r*_ of the multi-photon rate decreases, and it has the following relationship with 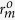,


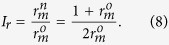


We draw *I*_*r*_ as a function of 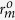 in Fig. 4(b), which shows that the better the laser source is, the larger the multi-photon rate increases. For instance, when 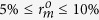, the multi-photon rate increases five to ten times. This can be easily detected and represents proof of the presence of Eve.

To decrease the probability of being detected, Eve can use the second strategy, i.e., inserting a spy photon in part of legitimate signals. Similarly, let 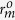 be the original multi-photon rate, and *n* be the number of signals in the *S*-sequence. We assume that Eve inserts a spy photon in a legitimate signal with probability *s*. After Eve’s attack, there are 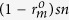 signals containing two photons. Half of these signals can be detected as multi-photons. Bob can detect 
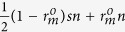
 signals that contain more than one photon. Therefore, the new multi-photon rate is


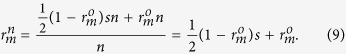


Indeed, as shown in Fig. 4(c), Eve’s strategy decreases the multi-photon rate. The fewer the spy photons that Eve inserts into legitimate signals, the lower the multi-photon rate that Bob can detect. However, if the original multi-photon rate is small enough, the multi-photon rate still increases significantly, and clearly, the increase rate *I*_*r*_ correlates with the original multi-photon rate and the probability that Eve inserts a spy photon into a legitimate signal:


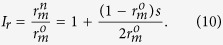


As shown in Fig. 4(d), when 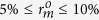, even though *s* is very low, let us say 10%, the multi-photon rate still increases by 45% and 95%. Therefore, using this defense strategy, our protocol can resist THA.

To analyze the security, it is critical to bound Eve’s accessible information. However, ref. 31 has already proposed a general method for calculating the eavesdropped information as a function of the attack detection probability. This method can be applied to schemes based on multiple qubits, qudit and GHZ states. Clearly, it also works for our protocol. Due to limited space, we will not discuss Eve’s accessible information here. See ref. 31 for details.

**Figure 5:**
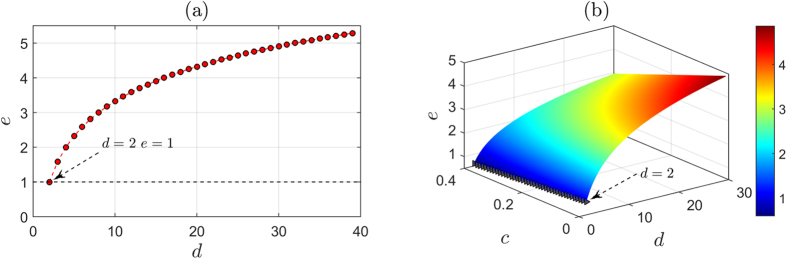
Efficiency analysis. (**a**) Efficiency *e* versus the dimensions *d*. The dashed line shows the theoretical efficiency of existing single photon based schemes. (**b**) Efficiency *e* versus the dimensions *d* and the proportion *c* of photons that used for security checking. The gray triangles indicate the efficiency of existing single photon based schemes with different *c*.

For efficiency, theoretically, a *d*-level system can carry log_2_*d* bit information. As shown in Fig. 5(a), a QSDC protocol achieves higher efficiency if it employs a higher dimensional system. Our protocol, therefore, reaches higher efficiency compared with existing single photon-based schemes. In practice, however, the legitimate users need to use part of the photons to check the security of the quantum channel. Thus, the efficiency not only correlates with the dimensions but also correlates with the proportion of photons used for security checking. Suppose Alice sends *n* photons to Bob. They use *c*% photons for security checking. That is, (1 − *c*)% photons are used for message transmission. Since each photon can carry log_2_*d* bits, a total [(1 − *c)n*] log_2 _*d* bit secret message can be transmitted. Therefore, the efficiency is


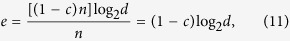


i.e., on average, each photon carries (1 − *c*) log_2 _*d* bit information. We plot the efficiency *e* as a function of the dimensions *d* and the proportion *c* of photons used for security checking in Fig. 5(b), where gray triangles label the efficiency of existing single photon-based schemes with different *c*. Clearly, with the same *c*, our protocol is more efficient.

As discussed above, the present protocol is secure and efficient in an ideal lossless channel. In a practical quantum channel, there are noise and loss that will threaten the security and decrease the efficiency of quantum communication. Similar to the protocols proposed in refs 7 and 14, our protocol is secure under conditions of weak noise. This is because in a low noise channel, Eve’s attack will increase either the error rate or the photon loss rate and can thus be detected. However, if the channel noise is sufficiently high, Eve can replace the quantum channel with a better one and hide her attack in the noise. In this case, Alice and Bob cannot detect Eve’s attack. To solve this problem, first, additional quantum error correction and privacy amplification steps are recommended^12^. Second, noiseless linear amplification (NLA)^32^ is also recommended for experimental demonstration. With a noiseless linear amplifier, it is possible to perform tasks such as purifying a lossy channel, distilling entanglement, etc. The concept of NLA was first proposed by Ralph and Lund in 2009^32^. Since then, many advancements have been achieved. For instance, Xiang *et al*. experimentally demonstrated the heralded NLA^33^. Kocsis *et al*. constructed a coherent two-mode amplifier to demonstrate the heralded NLA of a qubit encoded in the polarization state of a single photon^34^. Zhou and Sheng proposed a highly efficient recyclable amplification protocol that can be used to protect the single photon entangled state^35^. Yang *et al*. introduced a linear-optical amplification protocol to protect a single photon from photon loss^36^. Clearly, NLA is a powerful way to overcome the photon loss problem, and it has been employed in device-independent QKD^37^. It can also be deployed on QSDC and DSQC protocols against channel noise.

## Discussion

There are different types of DSQC protocols. In this section, we compare our protocol with four typical protocols. The first protocol is Li’s protocol^13^, which is based on a *d*-level quantum system^13^. To the best of our knowledge, this the first and only *d*-level single photon-based DSQC protocol. Since our protocol is based on data block transmission and order rearrangement techniques, we make a detailed comparison between our protocol and two similar protocols: one is Zhu’s protocol^14^, which is based on EPR pairs, and the other is Wang’s protocol^15^, which is based on two-level single photons. Finally, we compared our protocol with a typical GHZ-based DSQC protocol^19^, i.e., Yuan’s protocol.

In Li’s protocol, Alice first prepares a sequence of *d*-level single photons and encodes her secret message on part of these photons. The remaining photons act as decoy photons to check the security. Then, Alice sends these photons to Bob. After Bob receives these photons, Alice announces the positions and the states of the decoy photons. By measuring them, Bob can obtain the error rate of the transmission. If the error rate exceeds the threshold, they abort the transmission. Otherwise, Alice tells Bob the original states of the photons carrying the secret message. Bob can read out the secret message by measuring these photons with the same measuring basis as that chosen by Alice when preparing them. Similar to our protocol, Li’s protocol is based on *d*-level single photons. This makes it achieve higher feasibility compared with entanglement-based schemes and reach higher detection rate and efficiency compared with the two-level system-based protocol. Clearly, Li’s protocol is a one-way protocol. Compared with two-way schemes, since the photons in Li’s protocol only need to be transmitted from Alice to Bob, it can resist THAs and avoid the noise and loss arising from the backward transmission. However, compared with our protocol, Li’s protocol has a security weakness: Eve can hide her attack in channel noise. Suppose Eve evades security checking and obtains part of the legitimate photons. In Li’s protocol, since Alice needs to announce the original states of the photons carrying the secret message, Eve can deterministically read out the secret message encoded on these obtained photons. In our protocol, the original states of the photons are completely secret to others. Even though Eve evades all security checking, she does not know the original states of the obtained photons. All she can do is randomly select a basis and measure these photons. In this case, she only has a probability of 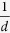 of selecting the correct basis. The probability would converge to 0 when *d* is large enough. That is, Eve can obtain nothing even if she evades security checking. Therefore, our protocol achieves higher security compared with Li’s protocol.

In Zhu’s protocol, Alice first prepares a set of EPR pairs and divides them into two partner-photon sequences. Then, she sends one sequence of the photons to Bob, who randomly chooses a sufficiently large subset of photons as a checking set and the rest as a message set. Bob encodes his checking message on the checking set and the secret message on the message set. Then, he disturbs the initial order of the photons and sends them back to Alice. After verifying that Alice has received all photons, Bob announces the position and the secret order of the photons carrying the checking message. By performing Bell measurements, Alice and Bob can decide whether Eve is online or not. If Eve is online, Bob terminates the communication. Otherwise, he publishes the secret order of the photons carrying the secret message. Alice can obtain Bob’s secret message by measuring these photons. Wang’s protocol is similar to Zhu’s protocol except that it uses a single photon instead of EPR pairs. In this protocol, Alice first prepares a set of photons in one of four different states and then sends them to Bob. Bob measures part of these photons to check the security. If the quantum channel is secure, with probability *c*, Bob encodes the checking message in the received photon and, with probability (1 − *c*), encodes his secret message in the photon. Bob disturbs the order of these photons and sends them back to Alice. After Alice announces that she has received these photons, Bob publishes the position of the photons carrying the checking message. Alice then measures these photons with the basis she used to prepared theses photons to read out the checking message. By comparing the checking message with Bob, Alice can calculate the error rate. If it exceeds the preset threshold, they abort the protocol. Otherwise, Bob publishes the position of the photons carrying the secret message. Alice measures these photons to obtain the secret message. For security, both Zhu’s and Wang’s protocols are based on data block transmission and order rearrangement techniques. However, since only two-level system are employed in these protocols, the detection rate is only 25%. Additionally, in Zhu’s protocol, only the security of backward transmission is verified. This further decreases the probability of detecting Eve’s attack. Moreover, these protocols cannot resist the THA (see ref. 5 for details). By using this attack strategy, Eve can obtain Bob’s full information without being detected. In our protocol, both the forward and backward transmissions are carefully checked. The detection rate of our protocol far outweighs the rates of these protocols; it converges to 100% if the dimension of *d* is large enough. Our protocol can also resist the THA because the multi-photon rate increases significantly, even though Eve inserts spy photons in small parts of legitimate signals. Therefore, our protocol is more secure than these protocols. For efficiency, similarly, suppose that legitimate users use *c*% photons to assess the security. Since Zhu’s protocol uses an EPR pair to transmit two bit information, the efficiency is 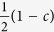. Clearly, the efficiency of Wang’s protocol is also (1 − *c*). Therefore, our protocol is more efficient than these protocols.

In Yuan’s protocol, Alice first prepares a set of GHZ state sequence and encodes the secret message on the second and third photons. Then, she takes the first photon from each triplet to form a single photon sequence. The second and third photons construct a photon pair sequence. Alice disturbs the order of the photon pairs and adds some decoy photons (each of the decoy photons is prepared in one of four different states) into it. She sends these photons to Bob and announces the positions and the states of the decoy photons after confirming that Bob has received these photons. By measuring the decoy photons, Bob can evaluate the error rate of the transmission. If the error rate exceeds the threshold, they abort this communication. Otherwise, Alice exposes the secret order of the photon pairs. Then, Alice measures her photons, and Bob performs Bell-basis measurements of the photon pairs. Alice publishes her measurement results to Bob via a classical channel. According to this information, Bob can obtain the secret message. Similar to Li’s protocol, Yuan’s protocol is a one-way protocol, which makes this protocol inherently resistant to THAs. However, since the security of the transmission is checked by measuring the decoy states, the detection rate of Yuan’s protocol is only 25%, and similar to Li’s protocol, if Eve evades the security check and obtains part of the photon pairs, she can deterministically read out the secret message encoded on these obtained photons after Alice announces the secret order of photon pairs and her measurement results. For efficiency, suppose the legitimate users use *c*% photons to check the security. Since Yuan’s protocol uses a GHZ state to transmit two bit information, the efficiency is 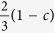. Clearly, our protocol is more secure and efficient than Yuan’s protocol.

To sum up, we propose a DSQC protocol using a single *d*-level system. It employs the data block transmission technique proposed in ref. 3 and the order rearrangement technique proposed in ref. 26 and uses a high-dimensional quantum system as the message carrier. These employed techniques guarantee the security of our protocol, and the high-dimensional quantum system enhances the security and increases the efficiency. Compared with entanglement-based schemes^14^,^16^,^17^,^18^,^19^, our protocol uses only sequential communication of a single qudit, which has huge advantages in feasibility and can be realized with current technology. Compared with existing two-level single photon-based schemes^4^,^15^, our protocol is more secure and efficient, and compared with one-way DSQC protocols^13^,^19^, our protocol achieves higher security. See Table 1 for details.

## Methods

Clearly, quantum memory is required to implement our DSQC protocol. Storage of quantum information is a key element in quantum information processing and quantum communication networks. Quantum memory has therefore attracted intensive study, and many theoretical solutions and experimental implementations have been proposed in recent years. For example, based on electromagnetically induced transparency, Lukin *et al*. reported an experiment in which a light pulse was trapped in a vapor of Rb atoms, stored for a controlled period of time and then released on demand^38^. Fleischhauer *et al*. introduced a quantum memory for light based on dark-state polaritons^39^. Hedges *et al*. realized a low-noise, highly efficient quantum memory for light using solid-state medium^40^. Specht *et al*. presented a quantum memory for polarization qubits based on a single trapped atom^41^. Recently, high-dimensional quantum memory has attracted more attention, as encoding photons with a spatial shape through higher dimensional states increases the capacity and security. In 2013, Ding *et al*. reported the first experimental implementation of a single photon stored in a cold atomic ensemble^42^. Although this work is a proof of principle, it makes an important step toward realizing high-dimensional quantum memory. In 2014, this research group reported a demonstration of quantum memory storing a photon encoded in a three-dimensional space^43^. In 2015, Zhou *et al*. presented a quantum storage of three-dimensional entanglement in a rare-earth-ion-doped crystal^44^. Additionally, in 2016, Ding *et al*. realized high-dimensional entanglement between distant atomic-ensemble memories^45^. This work confirmed the successful preparation of a state entangled in a seven-dimensional space. Clearly, some of these solutions can be employed as quantum memory to realize our protocol.

**Figure 6:**
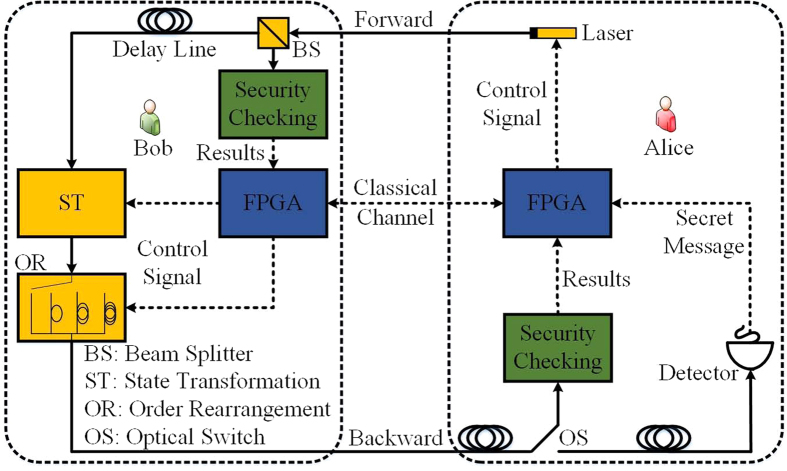
Implementation of our DSQC protocol.

Recently, Hu *et al*. demonstrated the DL04 protocol^8^ in an experiment using a delay line^10^. Similar to this work, our protocol can also be realized with a delay line. As shown in Fig. 6, Alice first prepares a set of photons and sends them to Bob, who uses a beam splitter to select a subset of photons to evaluate the security of the forward transmission. The remaining photons are stored in the delay line. If the error rate or the multi-photon rate is unreasonably high, Alice and Bob abort the transmission. Otherwise, Bob encodes the checking message and secret message on these photons. Then, he disturbs the initial order of these photons by sending them through different delay lines. When Alice receives these photons, she first stores them in a delay line. After Bob announces the position and the order of the photons carrying the checking message, she uses an optical switch to select photons carrying the checking message to evaluate the security of the backward transmission. The photons carrying the secret message are stored in another delay line. If the backward transmission is secure, Bob publicizes the rearranged order of the photons carrying the secret message. Then, Alice can use the same measuring basis as when she prepared the photon to measure it and read out the secret message. Clearly, our protocol can be realized with current techniques.

## Additional Information

**How to cite this article:** Jiang, D. *et al*. Deterministic secure quantum communication using a single d-level system. *Sci. Rep.* **7**, 44934; doi: 10.1038/srep44934 (2017).

**Publisher's note:** Springer Nature remains neutral with regard to jurisdictional claims in published maps and institutional affiliations.

## Figures and Tables

**Table 1:** Comparison between our DSQC protocol and typical DSQC protocols.

	Our Protocol	Ref. 13	Ref. 14	Ref. 15	Ref. 19
Based On	*d*-level SP	*d*-Level SP	EPR	2-Level SP	GHZ
Security Checking	F + B	One-Way	B	F + B	One-Way
Order Rearrangement	Yes	No	Yes	Yes	Yes
Detection Rate	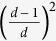	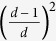	25%	25%	25%
Resistance to THA	Yes	Yes	No	No	Yes
Efficiency	(1 − *c*) log_2 _*d*	(1 − *c*) log_2 _*d*	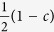	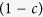	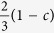

In this table, SP, F and B indicate single photon, forward transmission and backward transmission, respectively. *d* and *c* denote the dimensions and the proportion of photons that Alice and Bob used for eavesdropper checking, respectively.
